# Ubiquitous Creation of Bas-Relief Surfaces with Depth-of-Field Effects Using Smartphones

**DOI:** 10.3390/s17030572

**Published:** 2017-03-11

**Authors:** Bong-Soo Sohn

**Affiliations:** School of Computer Science and Engineering, Chung-Ang University, Seoul 06974, Korea; bongbong@cau.ac.kr; Tel.: +82-2-820-5843

**Keywords:** smartphone application, computer graphics, ubiquitous computing

## Abstract

This paper describes a new method to automatically generate digital bas-reliefs with depth-of-field effects from general scenes. Most previous methods for bas-relief generation take input in the form of 3D models. However, obtaining 3D models of real scenes or objects is often difficult, inaccurate, and time-consuming. From this motivation, we developed a method that takes as input a set of photographs that can be quickly and ubiquitously captured by ordinary smartphone cameras. A depth map is computed from the input photographs. The value range of the depth map is compressed and used as a base map representing the overall shape of the bas-relief. However, the resulting base map contains little information on details of the scene. Thus, we construct a detail map using pixel values of the input image to express the details. The base and detail maps are blended to generate a new depth map that reflects both overall depth and scene detail information. This map is selectively blurred to simulate the depth-of-field effects. The final depth map is converted to a bas-relief surface mesh. Experimental results show that our method generates a realistic bas-relief surface of general scenes with no expensive manual processing.

## 1. Introduction

Relief is a sculpting method whereby 3D shapes of objects are projected, compressed, and attached to a background plane. There are different types of reliefs depending on the degree of depth compression. Bas-relief is a special form of a relief in which 3D shapes are sculpted as shallow, highly depth-compressed elements. Bas-relief generation has wide applications in making objects such as commemorative medals, coins, souvenirs, and artistic sculptures for blind people.

3D printing technology is undergoing rapid development, and thus there is a need for algorithms that allow users to create bas-relief surfaces of scenes or objects. Many previous approaches for bas-relief generation focus on compressing the depths of 3D scenes while ensuring that their three-dimensional features remain clear [1,2,3,4]. However, obtaining 3D models of real scenes or objects is often difficult (or sometimes impossible) and time-consuming. 3D models reconstructed from real scenes are often inaccurate. Furthermore, scanning real scenes to make 3D models often requires expensive equipment.

Recently, smartphones have become ubiquitously available, and virtually every smartphone has a built-in digital camera. This represents an opportunity to use smartphones as 3D scanning equipment. Users can take photographs of any general scene with these cameras, including indoor or outdoor scenes with or without human subjects, and a multiview reconstruction algorithm [5] can be used to generate a depth map from the acquired photographs. The photograph and depth map can then be transmitted to a computer that can compute a bas-relief surface mesh based on these inputs. The bas-relief surface mesh can then be sent to a 3D printer to physically generate a bas-relief sculpture. This process can be initiated from anywhere because all the equipment (i.e., smartphones, desktop computers, and 3D printers) can be connected through the Internet. To implement this system, an automatic algorithm is necessary that can accept photographs captured by a smartphone and use them to generate a bas-relief surface.

There are three main difficulties in preparing digital bas-relief surfaces from photographs. First, a single photograph does not contain the depth information that is necessary for making a bas-relief surface. Although a stereoscopic camera can be used for generating depth maps, such specialized equipment is expensive and cumbersome, and thus not realistically available to most general users. Second, even if depth information is available, naive methods that linearly compress the range of the depth map often poorly restore the 3D shapes of features. Third, main objects and background objects can easily become intermixed in surfaces generated from complex scenes, presenting distractions that make it difficult to concentrate on the main objects. In the field of photography, depth-of-field (DOF) control is used to overcome this problem, allowing main objects to be displayed clearly and background objects to be displayed dimly. Similar DOF control can be applied to generation of bas-relief surfaces, but this possibility has not been addressed in methods previously proposed.

From this motivation, we propose a new method for bas-relief generation, which has following unique characteristics and advantages:

- **Ubiquitous**: our method takes as input photographs of any general scene, which can be captured anytime by using the built-in digital camera of an ordinary smartphone.
- **Realistic**: our method generates a realistic bas-relief surface, including restoration of visual details as well as overall depths of objects on the surface.
- **DOF**: our method generates a DOF effect on each surface based on a user-specified focal point and degree of DOF blurring.
- **Automatic**: our method does not require manual work from the user (e.g., region segmentation), excluding the selection of a few parameter values.

The proposed approach to construct relief surfaces comprises four main phases. Input is given as a set of sequential photograph images. In the first phase, a reference image and its depth map are obtained. In the second phase, a base depth map is constructed from the initial depth map. An adaptive histogram equalization method is used to enhance the local contrast of the depth map by locally elongating depth ranges that include many pixel values and shortening ranges that have few pixel values. To enhance the DOF effect, the adaptive histogram equalization algorithm also renders the main objects in greater depth contrast and the background objects in less. In the third phase, a map for detail relief is constructed. The reference image and the gradient of the reference image are processed to represent high-frequency details that will be carved into the base map. In the fourth phase, the base and detail maps are blended to construct a relief map. To simulate DOF effects, this relief map is selectively blurred according to the input depth-of-field parameter. This process improves the effect of emphasizing main objects in focus and de-emphasizing background objects in the resulting bas-relief surface. The final relief map is used to make a mesh describing the bas-relief surface. Experimental results presented herein show that our method easily generates a bas-relief that looks realistic and natural, using an ordinary smartphone and PC.

The remainder of this paper is organized as follows. After reviewing related papers in Section 2, the overall process of our method is explained in Section 3. A final bas-relief is prepared from composition of a low-frequency base relief and a high-frequency detail relief. Section 4 describes the techniques used for generating a depth map expressing the base relief, based on a depth-weighted adaptive histogram equalization method. Section 5 describes the techniques used for generating a depth map expressing the detail relief. Section 6 describes our method for combining the base and detail depth maps, incorporating DOF effects, and generating a surface mesh from the resulting depth map. Section 7 presents experimental results of the proposed method. Section 8 presents our conclusions.

A preliminary version of this paper appeared in [6]. We extend our previous work by proposing new algorithms for generation of base maps, detail maps, and depth-of-field effects with detailed description. We also added extensive test results including the results of 3D printing.

## 2. Related Work

Bas-relief has been an important sculptural technique used to render various types of objects such as gods, heroes, and natural scenes. A significant amount of research has been done to design computer algorithms to generate bas-relief surfaces in digital form that can be 3D-printed into physical bas-relief sculptures. Most of these algorithms can be classified into two groups, depending on the type of input: either 3D models or an image.

Cignoni et al. [1] introduced the problem of bas-relief and high-relief generation from 3D scenes and proposed a simple algorithm for depth compression. Bas-relief is represented as a depth map in which each depth value is the distance to the corresponding object point from the camera’s viewpoint. The depth map is then compressed such that the depth of the resulting surface is reduced to a user-specified range. The compressed depth map can be directly converted to a surface mesh. Weyrich et al. [3] improved the algorithm by adding a nonlinear depth compression method whereby the gradient of the depth map is preserved to express the shape with sufficient details while maintaining the shallow depth of the relief surface. Song et al. [7] and Kerber et al. [8] developed methods for restoring salient features based on unsharp masking. Ref. [3,8] utilized a High Dynamic Range (HDR) method for enhancing contrasts without increasing depth ranges. Kerber et al. [2] extended [3] to include a nonlinear scaling method and bilateral filtering for gradient decomposition. Sun et al. [9] used and improved the contrast-limited adaptive histogram equalization (CLAHE) method to enhance local contrasts in the depth map and emphasize salient features. Although the resulting algorithm is simple, its results are comparable to or sometimes better than those in [3] depending on the parameter settings and test data. Zhang et al. [4] developed a method whereby the input object is decomposed into a low-frequency base mesh and high-frequency detail features, and a map is generated having a height domain. Adaptive linear compression is used to enhance the visual details.

Most of the above methods create a depth map of the input 3D scene that is projected from a given viewpoint, and include linear or nonlinear depth compression methods for creating the resulting bas-relief. However, these methods require inputs in the form of 3D models, which are often difficult and time-consuming to prepare. For this reason, algorithms have been designed for bas-relief generation based upon inputs in the form of images. The specific types of image inputs can include rubbing images generated by brick and stone relief [10] or painting images [11,12]. However, the most common form of the input image is a photograph, which can be obtained anywhere by using a smartphone. Since images usually do not have accurate depth information, it is necessary to have effective methods available to estimate depth information while also expressing the visual details of objects in the input image.

Li et al. [10] described a new method for restoring brick and stone relief from rubbing images. They constructed separate height maps for low-frequency base relief and high-frequency details and then combined them to generate the realistic relief. Their results showed that the direct application of this method to a general photograph may result in unnatural reliefs. Wu et al. [13] developed a method that generates bas-reliefs from photographs of human faces, based upon a neural network that is trained using images. The method proposed in [14] is similar to that of our present work; it takes input in the form of a general image, which could include a photograph, and generates a bas-relief based on the image. The method of [14] constructs the depth map of a base mesh based on region segmentation from the input image; however, this segmentation requires time-consuming manual work. Contrastingly, our work is based on the use of photographs to estimate depth, an alternative that takes advantage of smartphone capability. In our previous work [15,16], we described a method that uses facial feature detection and enhancement for bas-relief generation from a photograph of a human face.

The depth-of-field effect is an important concept in photography. Although the DOF effect has not been applied in the area of bas-relief generation, various algorithms for 3D rendering with DOF effects have been developed. Ref. [17] introduced a realistic camera model instead of the traditional pinhole camera model used for 3D rendering with DOF effects. These concepts can be implemented with OpenGL for graphical rendering [18]. A graphics processing unit (GPU) acceleration method has been developed for real-time rendering with lens blur effects [19]. One of the main contributions of the present work is to apply DOF effects for realistic bas-relief generation.

## 3. Overview

**Figure 1:**
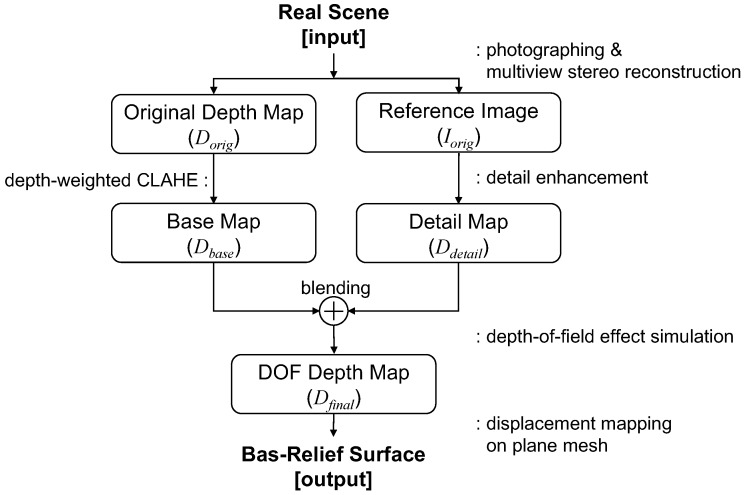
Overall flow of the proposed method.

The overall flow of our method is depicted in Figure 1. Table 1 describes various image maps used in the figure. Our approach consists of four main parts: (i) input image and depth map acquisition; (ii) generation of a base map; (iii) generation of a detail map; and (iv) generation of bas-reliefs with DOF effects.

First, an input is given in the form of a sequence of photographs. The first image of the sequence in grayscale format becomes a reference image $I_{orig}\left(x,y\right)$ that will be converted into a bas-relief surface. A depth map $D_{orig}\left(x,y\right)$ can be obtained from the sequence of photographs by using a vision algorithm called multiview stereo reconstruction [5]. The photograph acquisition and processing of vision algorithms can be carried out by using a smartphone application called Google Camera [20] (ver 3.2.042, Google Inc., Mountain View, CA, USA). The processing carried out by the Google Camera application is summarized as follows. First, the 3D positions and orientation of the camera and the image features are computed and tracked for each image in the sequence. Next, using the multiview stereo algorithm, 3D locations of the feature points are estimated and a 3D model of the scene in the reference image is constructed, and this model is used to create a depth map of the scene.

Second, a base map $D_{base}\left(x,y\right)$ representing the depth map of a low-frequency base relief is computed. The initial input depth map $D_{orig}\left(x,y\right)$ is nonlinearly compressed to have a thin range specified by the user. In this depth compression process, adaptive histogram equalization is applied to ensure that each object in the scene has enough local contrast. In particular, our method gives depth weight when limiting pixels in each histogram bin so that main objects that are near or within the area of focus can have more contrast in the depth map, whereas background objects will have less contrast. The degrees of contrast adjustment and shape blurring can be controlled by means of user parameters.

Third, a detail map $D_{detail}\left(x,y\right)$ is computed that represents the depth map of high-frequency detail relief. For this purpose, we use an original grayscale image $I_{orig}\left(x,y\right)$ and an edge map E(x,y). The edge map can be computed from the input image by applying an existing edge extraction algorithm. We propose filtering algorithms that adapt the input image and edge map into a form that is appropriate for representing the detail map, which gives the resulting surface more realism.

As the final step, the base and detail maps are blended to form a single depth map. We apply per-pixel post-processing to create DOF effects in the depth map. As a result, background objects that are out-of-focus will look blurry, whereas foreground objects will look sharp. The degree of DOF effects and the selection of in-focus objects can be specified by the user. The resulting depth map $D_{final}\left(x,y\right)$ is directly converted into a bas-relief surface mesh.

Zeng et al. [14] proposed a similar method that generates bas-relief from a photograph. They introduced the equation

$$D_{final}\left(x,y\right)=\alpha \cdot D\left(x,y\right)+\beta \cdot I\left(x,y\right)+\gamma \cdot G\left(x,y\right),$$

where D(x,y) is a depth map estimated from input image I(x,y); G(x,y) is a gradient magnitude map of I(x,y); and *α*, *β*, and *γ* are user-controlled coefficients.

Differently, our method expresses the final relief map as follows:

$$D_{final}\left(x,y\right)=DOF\left(\alpha \cdot D_{base}\left(x,y\right)+D_{detail}\left(x,y\right)\right),$$

$$D_{detail}\left(x,y\right)=\beta \cdot I_{contrast}\left(x,y\right)+\gamma \cdot E\left(x,y\right),$$

where DOF is a post-processing per-pixel operation that generates DOF effects on a given depth map, and $I_{contrast}$ is an image that is generated by applying local contrast enhancement to $I_{orig}$. An edge map E(x,y) is used to emphasize salient features of objects. Depending on the type of edge extraction algorithms used, the result will have different effects. Our main improvement compared to the work of Zeng et al. [14] is (i) to make DOF effects on the resulting bas-relief surface; (ii) to automate the process of computing the base relief map $D_{base}$ without region segmentation, which would require a significant amount of manual work; (iii) to use $I_{contrast}$ instead of $I_{orig}$ to highlight local details; and (iv) to use a general edge map E(x,y) instead of G(x,y).

## 4. Generation of Base Map

The original depth map $D_{orig}\left(x,y\right)$ stores depth values that represent rough distances from the camera to object points. Although the depth values are not accurate enough to be used for reconstruction of fine geometry, they are adequate to represent the overall shapes of objects in the input photograph. Therefore, we use a depth map $D_{orig}\left(x,y\right)$ to construct a low-frequency base relief. The main problem of the original depth map $D_{orig}\left(x,y\right)$ is that it does not have enough contrast to express convex and concave features of objects. Naive application of $D_{orig}$ to represent $D_{base}$ will easily produce flat object surfaces in the resulting bas-relief surface. We propose the use of depth-weighted CLAHE (contrast limited adaptive histogram equalization) to enhance local contrasts for each pixel while de-emphasizing background objects for DOF effects.

Our algorithm for contrast enhancement of the depth map extends the adaptive histogram equalization method described in [9,21]. This extended algorithm is used to make a uniform intensity distribution, which has the effect of improving overall contrasts in an image. We first summarize the description in [9] of the adaptive histogram equalization method and then propose our method by describing how to modify the equations to limit the level of contrast according to depth values. Suppose the input depth map is $D_{orig}\left(x,y\right)$. *B* bins are created and each depth value is assigned to its corresponding bin. This yields a histogram $\left\{h_{1},...,h_{B}\right\}$ in which $h_{i}$ represents the number of depth values that belong to bin $b_{i}$. The cumulative histogram $\left\{c_{1},...,c_{B}\right\}$ is then given as

$$c_{i}=\sum_{j\leq i}h_{j}.$$

During the histogram equalization process, each depth map value is transformed into a new value in a uniformly distributed way. As a result, global contrast in the image is enhanced. Suppose the depth value $D_{orig}\left(x,y\right)$ was assigned to bin $b_{i}$. Then, the transformed depth value $D_{orig}^{\prime }\left(x,y\right)$ will be as follows:

$$D_{orig}^{\prime }\left(x,y\right)=\frac{c_{i}-c_{1}}{c_{B}-c_{1}}\cdot D_{max}^{\prime },$$

where $D_{max}^{\prime }$ is specified to be the maximum depth value of the output so that the depth range is $\left[0,D_{max}^{\prime }\right]$. This transformation has the effect of enhancing global contrast in the input image. The above process can be improved by performing adaptive histogram equalization, in which histogram equalization is conducted locally within the neighborhood of each pixel point and the output value for that pixel is determined based on the local histogram. As a result, local contrast in the image is enhanced, thereby emphasizing local features and details.

Local cumulative histogram for a pixel point (x,y), $C\left(x,y\right)=\{c_{1}\left(x,y\right),...,c_{B}\left(x,y\right)\}$, is calculated as follows:

$$c_{i}\left(x,y\right)=\sum_{j\leq i}h_{j}\left(x,y\right).$$

If a pixel point (x,y) belongs to an *i*-th bin $b_{i}$, the new depth value $D_{orig}^{\prime }\left(x,y\right)$ is calculated as follows:

$$D_{orig}^{\prime }\left(x,y\right)=\frac{c_{i}\left(x,y\right)-c_{1}\left(x,y\right)}{c_{B}\left(x,y\right)-c_{1}\left(x,y\right)}\cdot D_{max}^{\prime }.$$

In this adaptive histogram equalization (AHE), the local histogram of all bins is calculated for each pixel point (x,y) and contributes to calculating the new depth value $D_{orig}^{\prime }\left(x,y\right)$. This local processing enhances contrasts and features for each local object in the result image.

Although adaptive histogram equalization enhances local contrast, pure application of AHE often causes intensity distortion by over-enhancing local contrast. This problem easily occurs when pixel intensities in a window are mostly distributed within a very narrow range of values. To make the image look more natural and obtain balance of contrast, a modification of AHE, called contrast-limited AHE (CLAHE), is often applied to limit the level of contrast. In CLAHE, a threshold value *θ* is specified such that elements in the overly accumulated bin that have an element number ratio bigger than *θ* are cut and uniformly redistributed among other bins.

An existing CLAHE method applies the uniform threshold value *θ* to limit the maximal size of each bin. In our method, we propose to reflect depth information by applying different thresholds Θ(x,y) depending on the depth value $D_{orig}\left(x,y\right)$ at each point (x,y). The purpose of using the different threshold values is to obtain DOF effects such that foreground objects have high contrast and background objects have relatively low contrast. Our depth-weighted CLAHE uses two user-specifiable parameters: the reference threshold value *θ* and the degree of threshold difference *k*; *k* of zero means that the same threshold is applied to each point regardless of depth, whereas high values of *k* mean that foreground objects having high depth values will have high threshold values for limiting contrast (i.e., high local contrast) and vice versa. We assume that the range of *k* is 0≤k≤1:

$$\Theta \left(x,y\right)=k\cdot \theta \cdot \left(RankRatio(\right|D_{orig}\left(x,y\right)-focalDepth|)-1)+\theta ,$$

where (x,y) is a pixel index, D(x,y) is the depth value at the point (x,y), and RankRatio(X) is the rank of *X* divided by the number of all possible values of *X* in the AHE window at (x,y) (0≤RankRatio(X)≤1).

**Figure 2:**
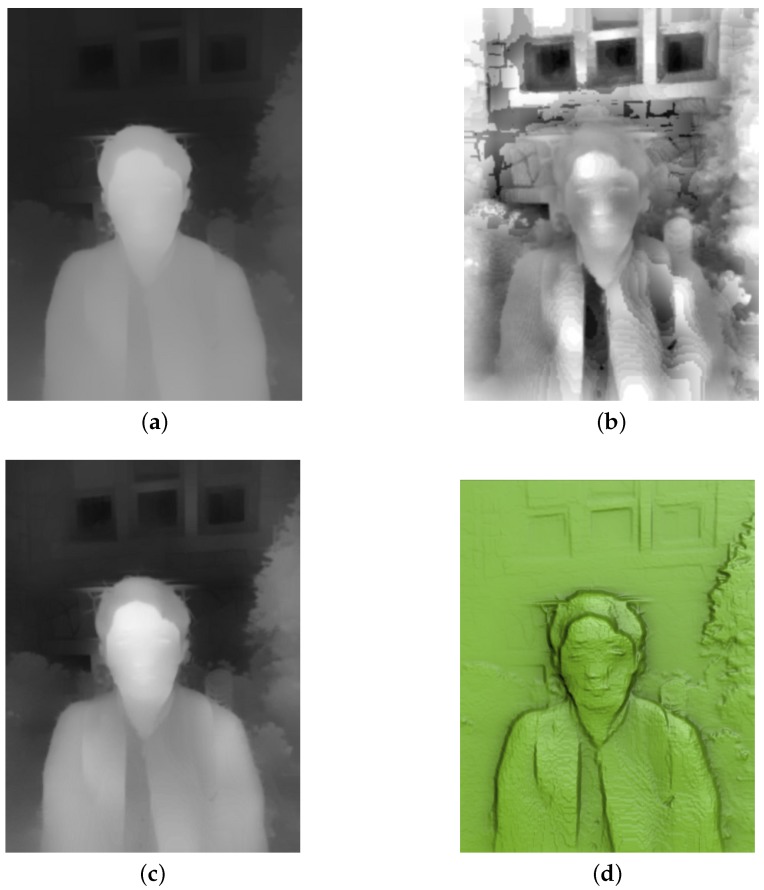
Results of low-frequency base relief generation from input depth map. (**a**) original depth map; (**b**) depth map adjusted using AHE; (**c**) depth map adjusted using the proposed method (i.e., depth-weighted CLAHE); and (**d**) relief surface of (**c**).

Figure 2a shows an example input depth map. The depth values represent overall depth patterns of each object in the scene. However, they lack detail features and do not have sufficient accuracy. To enhance the detail features, AHE can be applied to the input depth map. However, pure application of AHE may cause unrealistically high contrasts as shown in Figure 2b. In order to obtain natural and balanced contrast levels while enhancing local contrasts, CLAHE can be applied. We improve it by using the proposed depth-weighted CLAHE to give less contrast to out-of-focus background objects as shown in Figure 2c,d.

## 5. Generation of Detail Map

Because the depth map acquired by the smartphone camera application usually does not represent details precisely (see Figure 2), we need to generate a map that represents high-frequency details. This map is composited with the base relief map to yield a realistic relief. As similarly proposed in [14], the detail map can be expressed as a combination of an edge map and an intensity map. The intensity map I(x,y) is an original input grayscale image. The edge map E(x,y) consists of pixels that have high intensities in edge areas in the input image. We construct an initial edge map based on a Sobel operator. This map can then be smoothed for further operations.

The Sobel operator extracts edges by approximating the magnitude of gradients G(x,y) from the input image. The basic Sobel operator uses two 3×3 matrices, $M_{x}$ and $M_{y}$, that are convolved with an input image to compute the gradients. $M_{x}$ and $M_{y}$ are used for computing the horizontal and vertical components of the gradients, respectively:

$$M_{x}=\left(\begin{array}{ccc} -1 & 0 & +1\\ -2 & 0 & +2\\ -1 & 0 & +1 \end{array}\right),M_{y}=\left(\begin{array}{ccc} -1 & -2 & -1\\ 0 & 0 & 0\\ +1 & +2 & +1 \end{array}\right),$$

$$G_{x}=M_{x}*I,G_{y}=M_{y}*I,$$

where the operator * represents two-dimensional convolution.

In Sobel filtering, an edge map can be obtained based upon the gradient magnitude that is computed for each pixel according to the following equation:

$$G=\sqrt{{G_{x}}^{2}+{G_{y}}^{2}}.$$

**Figure 3:**
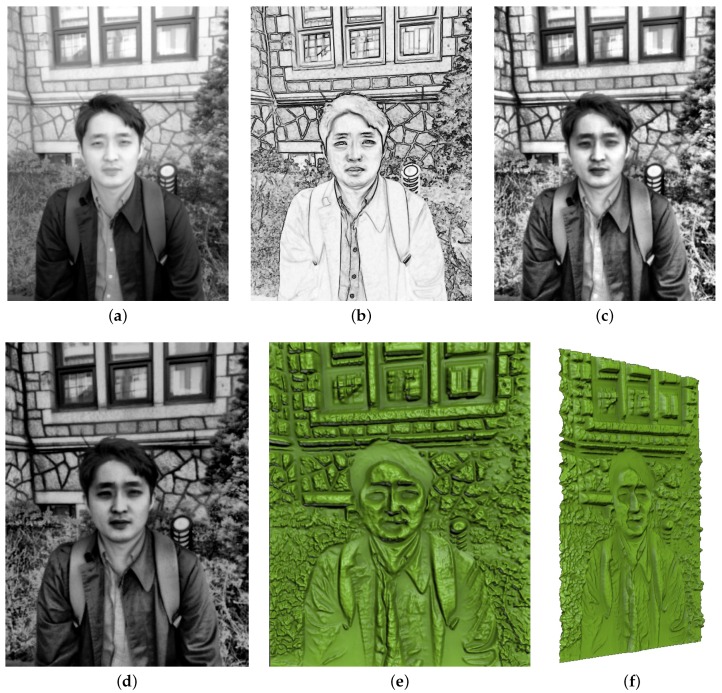
Results of high-frequency detail relief generation. (**a**) input grayscale image; (**b**) edge map; (**c**) image map with local contrast enhancement; (**d**) detail map (blending of (**b**,**c**)); (**e**) bas-relief surface of (**d**); and (**f**) (side view of (**e**)).

The edge map computed by applying the basic Sobel operator is shown in Figure 3b. As shown in the figure, the result clearly captures important features and details. However, it also contains a great deal of noise, which would result in a bumpy surface when converted to a relief surface. To reduce this bumpy effect while preserving salient edge features, we apply a selective Gaussian smoothing method. The original Gaussian blurring method smooths an input image by performing center-weighted averaging of neighbors with a Gaussian kernel function for each pixel. This method has the drawback of reducing sharp features that often represent salient object boundaries. The selective Gaussian method performs averaging of only the neighbors that have intensity differences below a given threshold value *τ*. Because pixel intensities often rapidly change over object boundaries, the boundary pixels will not be blurred.

The Gaussian kernel function used for image convolution is expressed as follows:

$$S\left(x,y\right)=\frac{1}{2\pi \sigma^{2}}e^{-\frac{x^{2}+y^{2}}{2\sigma^{2}}},$$

where *σ* is the standard deviation of the distribution. Discretization of the continuous Gaussian function is necessary to represent it as n×n matrix *L*. The new edge map where selective Gaussian blurring is applied is expressed as E(x,y)=G(x,y)*L. The user can control the range of blurring and the selection of neighbors for blurring by specifying the parameters *σ* and *τ*.

The detail map can be also specified by using the original image $I_{orig}$. The brightness value of $I_{orig}$ can be converted to a depth value to naturally represent surface textures on the bas-relief. Although brightness values give important clues for estimating depth level, the pattern of brightness values is often very different from the pattern of depth values. Therefore, direct linear mapping from brightness to depth values does not generate a natural-looking surface. For this reason, we apply an adaptive histogram equalization method to the original image to enhance local contrast levels. The resulting image $I_{contrast}$ contains information on local contrast, thereby showing surface textures while suppressing global contrast that can generate wrong depth to surface area.

We blend E(x,y) and $I_{contrast}\left(x,y\right)$ to construct a detail map $D_{detail}\left(x,y\right)$. Figure 3 shows an example of such a detail map and its relief surface. As shown in Figure 3e,f, the relief surface captures fine details of the input photograph, but it lacks overall depth information, making the main objects (i.e., the human subjects) look sunken. Therefore, the detail map needs to be blended with a base map to obtain a realistic relief surface.

## 6. Generation of Bas-Relief Surface with DOF Effects

**Figure 4:**
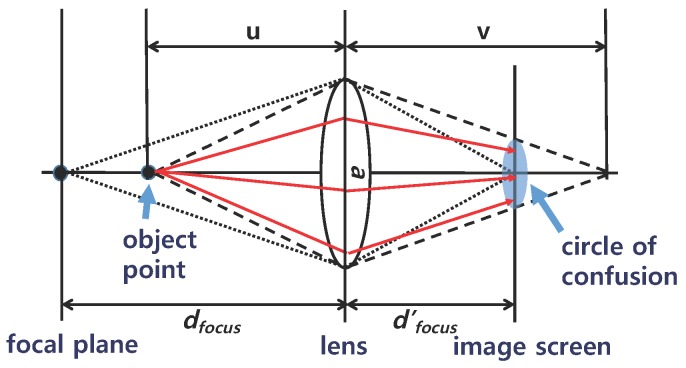
Thin lens model.

To further improve the out-of-focus effect, it is desirable to blur background objects. We simulate a thin lens camera model [17] for obtaining realistic DOF effects as shown in Figure 4. In this model, multiple rays are cast from an object point through a lens and projected onto an image plane behind the lens. If the object point is on a focal plane, all the multiple rays are mapped to single points on the image plane. If the object point is not on the focal plane (i.e., is out of focus), the rays shot from that point are distributed over a circular area on the image plane, making objects that are out of focus look blurry in the resulting image. This circular area is called the circle of confusion (CoC).

The computation of a CoC diameter, $CoC_{diameter}$, is described using the following lens equation [17]:

(1) $$\frac{1}{u}+\frac{1}{v}=\frac{1}{f},$$

where *u* is the distance between an object point and the lens, *v* is the distance between the focal point and the lens, and *f* is the focal length of the lens, as shown in Figure 4. Let *a* be the diameter of the lens (i.e., aperture size), $d_{focus}^{\prime }$ be the distance between the image plane and the lens, and $d_{focus}$ be the distance between the focal plane and the lens. Following the properties of similar triangles, we get

(2) $$CoC_{diameter}=\frac{a|v-d_{focus}^{\prime }|}{v}.$$

Using Equations (1) and (2), we get

(3) $$CoC_{diameter}=a\left|\left(\frac{1}{f}-\frac{1}{u}\right)\cdot d_{focus}^{\prime }-1\right|.$$

To apply the DOF effects to our depth map, we adopt a per-pixel approach by identifying and blurring the CoC of each pixel point as a post-process. At each pixel, the amount of blur depends on the depth. Since objects can have complex shapes, each pixel can have a different depth and hence a different amount of blurring. We use a filtering kernel with a variable size that depends on depth values to approximate the circle of confusion for each pixel.

The algorithm that simulates the DOF effect is described as follows. The inputs of the algorithm are the depth map $D_{orig}\left(x,y\right)$ and the relief map R(x,y) that is the result of blending the base and detail relief maps. For each pixel, we apply a blur filter whose size is determined according to the depth value and accumulate its contribution to the neighboring pixels. Thus, the intensity of each pixel is distributed to the neighboring pixels within its calculated CoC diameter. The data is accumulated into a new relief map $D_{final}\left(x,y\right)=DOF\left(R\left(x,y\right)\right)$, which is the final depth map for relief surface generation.

The light distribution [18] of a pixel representing an object point over a CoC can be modeled by using a kernel filter. We use a method that evenly distributes the intensity of light over a circular area. This uniform distribution method is simple but simulates the DOF effect with reasonable accuracy. In this method, the intensity of each pixel (x,y) is uniformly distributed onto the adjacent pixels in CoC. For the uniform distribution, the intensity of each object point needs to be divided by the area of the CoC and assigned to the adjacent areas in the CoC. This can be implemented by dividing the pixel intensity by the number of adjacent pixels and accumulating it to each of the adjacent pixels within the CoC to yield the output image $image_{output}$. This process can be modularized as follows and used for post-processing of each pixel.

1: Function $CoCLightDistribution(CoC_{diameter}$, *x*, *y*, $image_{input}$, $image_{output})$
2: clear $image_{output}$ with zero;
3: $r=CoC_{diameter}/2$;
4: pixelCount← number of adjacent pixels within CoC of (x,y);
5: **for** each (row,col)∈[x−r,...,x+r]×[y−r,...,y+r] **do**
6:   **if** $\left(\sqrt{{\left(row-x\right)}^{2}+{\left(col-y\right)}^{2}}\leq r\right)$ **then**
7:    $image_{output}\left[row,col\right]+=image_{input}\left(x,y\right)/pixelCount$;
8:   **end if**
9: **end for**

We use the function CoCLightDistribution to perform per-pixel intensity distribution over the CoC. Because the value in the depth map does not represent the exact distance to an object in the original image, it is difficult to compute the exact CoC. Instead, we perform an approximate computation for Equation (3) by increasing the CoC in proportion to the distance from the given depth value to the in-focus depth value. For a point that is too far from the focal plane, the CoC can be approximated as a constant value. The above approximation is expressed as $CoC\;=\;\mu |D_{orig}\left(x,y\right)-focalDepth|$, where focalDepth and *μ* are user-specifiable parameters for focal depth and the degree of blurring, respectively.

1: **for** each pixel (x,y) **do**
2:   $CoC_{diameter}\leftarrow \mu \left|D_{orig}\left(x,y\right)-focalDepth\right|$
3:   $CoCLightDistribution(CoC_{diameter},x,y,input,output$)
4: **end for**

The above post-processing blur techniques often cause a leaking problem. This problem happens because the distribution of background objects affects the pixels from any in-focus foreground objects that are nearby, due to the large filter size. To solve this problem, our method does not count samples that can contribute to such leaking. If the depth value of a pixel is close to the focus depth value, which means that the pixel is in focus, the pixel is in front of an adjacent pixel that is blurry, and the pixel should not contribute to the per-pixel intensity distribution.

**Figure 5:**
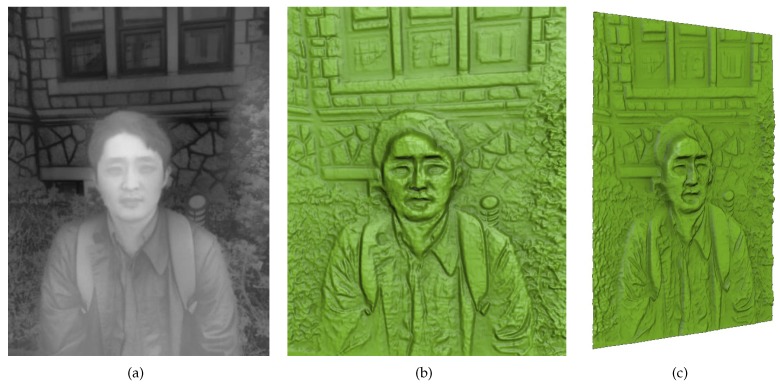

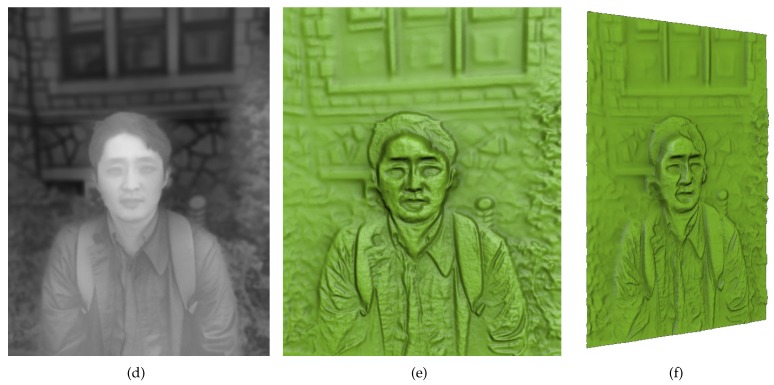
Results of relief generation with DOF effects. (**a**) relief depth map with no DOF effect; (**b**) relief surface of (**a**); (**c**) side view of (**b**); (**d**) relief depth map with DOF effect; (**e**) relief surface of (**d**); and (**f**) side view of (**e**).

Figure 5a,b show results of computing a relief map by blending a base map and detail map and constructing a surface from the relief map. The side view of this relief surface demonstrates that both overall depth and fine details of objects are well expressed on the surface (Figure 5c). We can observe that the foreground object (i.e., human subject) is protruded to express its shape with a shallow depth range. Figure 5d–f show the result of our method that generates DOF effects. The input scene is rather complex and contains many small features of background objects, which may distract the viewer. By blurring the out-of-focus background objects, we can obtain a relief surface that allows the viewer to concentrate on the main object. Users can generate different DOF effects by specifying the focal depth and the degree of blurring.

## 7. Experimental Results

We implemented and tested our method on an ordinary smartphone (Samsung Galaxy S3) and a desktop PC equipped with a 4.0 GHz Intel CPU and 16 GB of main memory. We obtained input photographs and a depth map by using the Google Camera smartphone application. Our implementation was based on MATLAB (R2013a, Mathworks, Natick, MA, USA) and C++ coding of the proposed algorithms. The proposed algorithms generate a depth map as output. We used 3ds Max software (2016, Autodesk, San Rafael, CA, USA) to convert the depth map to the resulting bas-relief surface by utilizing the function of displacement mapping and to render the surface. The 3D printer we used for bas-relief production is Ultimaker 2 (Ultimaker, Geldermalsen, Netherlands). It uses the Fused Deposition Modeling (FDM) method that prints by melting a plastic filament to produce the 3D object result. Ultimaker 2’s max layer resolution is 20 microns, xy and *z* positioning precision is 12 microns and 5 microns, respectively, and nozzle diameter is 0.4 mm.

Table 2 summarizes information on the test datasets and their execution time. Real scenes of high complexity and having different characteristics (e.g., indoor/outdoor, human/building/plamodel) were captured in the input photographs. The acquired input images were sent to the PC and processed to create bas-relief surfaces. The processing time was proportional to the size of the dataset. The total execution time was less than 20 seconds. Considering that our target application does not require real-time speed, this speed is acceptable for practical use.

**Figure 6:**
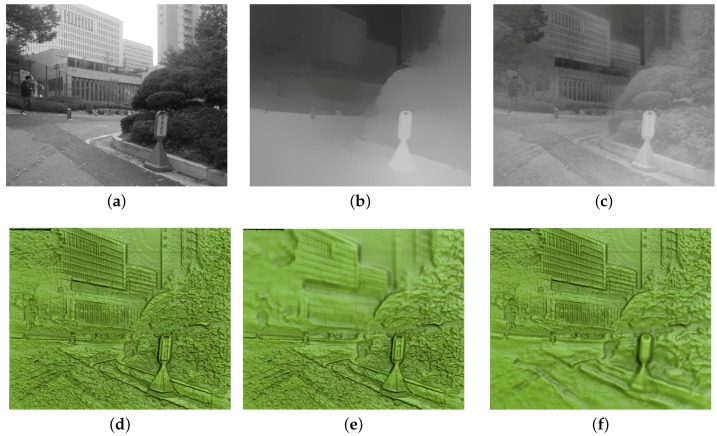
Results of bas-relief generation with DOF effects from the building data where different focus points were tested. (**a**) original image; (**b**) original depth map; (**c**) relief depth map; (**d**) relief surface with no DOF; (**e**) relief surface with close focus point; and (**f**) relief surface with far focus point.

Figure 6 shows the results for the building data. Figure 6a,b, respectively, show an input grayscale image and its depth map. Based on this input, our method computed the base map and detail map, and blended them into a relief depth map as shown in Figure 6c. To simulate DOF effects, we performed per-pixel post-processing to compute and blur the circle of confusion, according to a focal depth value given as a user input. Depending on the input focal depth values, we can obtain bas-reliefs with various DOF effects. As shown in Figure 6d–f, we can generate a bas-relief surface with no DOF effect, or with a DOF effect where the focal depth value is either close to or far from the camera. Adding the DOF effect makes in-focus objects look sharp and clear while other objects look blurry and less salient, allowing the viewer to concentrate on the main objects. Because we used both base and detail maps, the viewer can also perceive the overall depth of the objects as well as detail features in the bas-relief surface. However, the resulting surface included an incorrect representation of depth, demonstrating a weakness of our method. This phenomenon can be noticed in the sunken area of tree leaves located in the upper left parts of Figure 6c,d. This incorrect depth estimation was caused by using pixel intensity values for depth estimation. In spite of sacrificing the accuracy, we can obtain visual realism in the result. Since adaptive histogram equalization enhances local contrast, the level of image noise is also enhanced after the equalization process. The image noise often makes the resulting relief surface unnecessarily bumpy. In our experimentation, we applied a basic smoothing method to intermediate depth maps in order to reduce the noise problem.

**Figure 7:**
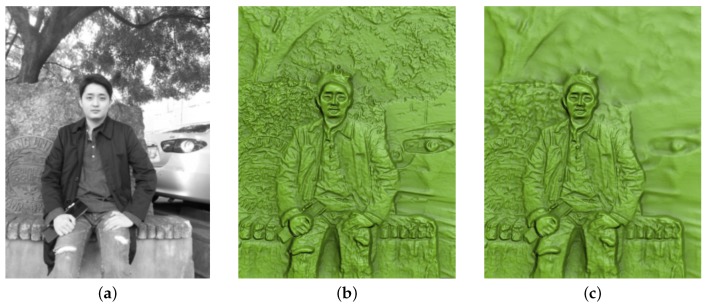
Results of bas-relief generation with DOF effects from the man2 data. (**a**) input image; (**b**) relief surface with no DOF effect; and (**c**) relief surface with DOF effect.

Figure 7 shows the result of testing how our method handles an outdoor scene including a human subject. The scene captured in the input photograph was highly complex, consisting of the human subject as the main object as well as trees, stones, and a car. Because trees and stones have many small features, the direct application of bas-relief generation without any DOF effects yielded many background details (i.e., trees and stones) on the resulting surface (Figure 7b). Therefore, a viewer of this surface might become distracted and have difficulty concentrating on the main object. Our method can generate DOF effects as a post-process such that the main objects located in the given focal area are unmodified and other background objects that are distant from the focal depth are blurred. The size of the blurring area is in proportion to the depth difference from the focal point. The result of applying these DOF effects is shown in Figure 7c. While the sharpness of the human subject is maintained, the stones located just behind the human subject are mildly blurred and the trees and the car that are positioned in the deep background are widely blurred.

**Figure 8:**
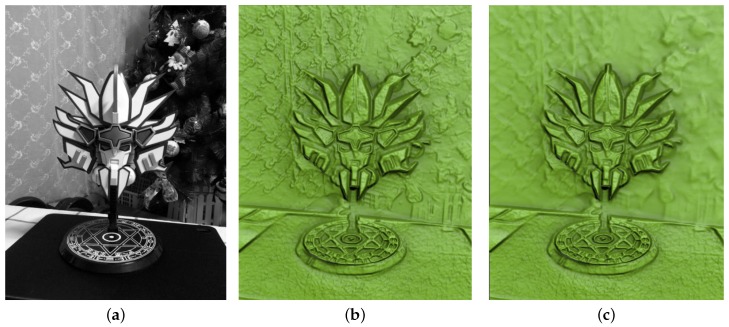
Results of bas-relief generation with DOF effects from the plamodel data. (**a**) input image; (**b**) relief surface with no DOF effect; and (**c**) relief surface with DOF effect.

Figure 8 shows the result of testing whether our method can appropriately handle the input of an indoor scene with ordinary goods. It contains a plamodel as the main object, a table, a curtain, and interior decorations. The user must specify the focal depth to specify the main object that is in focus. Because the background curtain and decorations have complex patterns, the main object does not look salient when no DOF effect is applied, and this makes it difficult for the viewer to sense the depth difference from the resulting surface (Figure 8b). After performing DOF processing, we obtained a result in which the main object looks salient as a result of blurring the patterns of the background curtains and decorations (Figure 8c).

**Figure 9:**
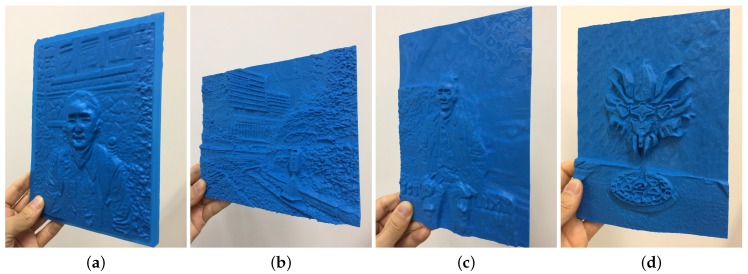
3D printed results generated from (**a**) man1, (**b**) building, (**c**) man2, and (**d**) plamodel data.

**Figure 10:**
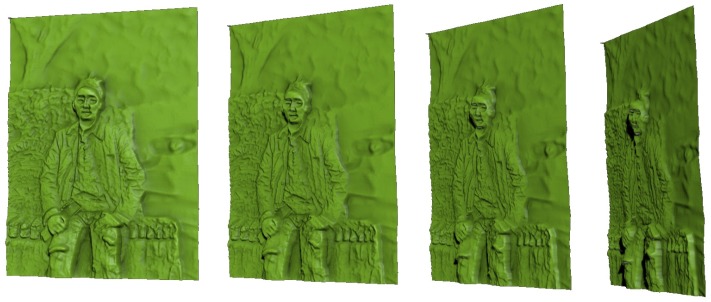
Side views of the bas-relief result generated from man2 data.

Figure 9 shows examples of 3D-printed models generated by using our method. Figure 10 shows side views of bas-relief surfaces prepared from the man2 data, where we can better see the depth of each point on the surface. The objects in the scene have the following order of increasing depth: human subject, stones, trees, and car. Figure 9 demonstrates that this order is maintained on the resulting bas-relief surface. Within the representation of the human subject, the protrusions of the legs, hands, and face are well expressed. Since the background trees and the car are relatively far from the focal point, they are blurred and are given only slight thickness for the purpose of DOF effects. Incorrect depth estimation was noted in the hair region of this image. This occurred due to inaccurate estimation of the initial depth map $D_{orig}$. It is expected that this problem could be alleviated by improving the depth map estimation algorithm. Objects that contain small features such as tree leaves and spotty patterns on stones were represented with high-frequency intensity changes in the input image. This characteristic was expressed as bumpy surfaces in the resulting bas-reliefs. The quality of 3D-printing results is rather low in terms of visual detail preservation compared to the rendering results of the bas-reliefs. This is mainly due to the insufficient printing precision of the 3D printer. The preservation of visual details in the results is expected to be improved as the 3D printer technology gets more developed.

Results of our method can be geometrically and topologically inconsistent with the original scene because of inaccurate depth estimation in our algorithms. On the other hand, our approach is highly practical in that the system works automatically and generates visually realistic results. Since the quality of bas-relief results involves artistic and aesthetic factors, the quantitative evaluation of performance in terms of accuracy becomes a challenging problem that is planned as our future work.

## 8. Conclusions

We have described a new method that creates a digital bas-relief surface from photographs. To improve visual realism and allow the user to concentrate on a main object in the scene, the method includes DOF effects based upon a user-specified focal point and degree of blurring. A depth map can be obtained from the input using an ordinary smartphone camera and an installed smartphone application. The depth map and input image are processed to build a base map representing the overall depth of the objects and a detail map representing high-frequency details. Both maps are composited and post-processed to generate a realistic bas-relief surface with DOF effects. The main advantage of our approach over previous methods that take 3D models as input is its ability to be applied ubiquitously and automatically. This approach allows any natural scene encountered in our daily life to be made into a bas-relief surface using an ordinary smartphone, without time-consuming manual work such as region segmentation. The resulting bas-relief surface can be rendered as a real object by means of 3D printing.

## Figures and Tables

**Table 1:** Description of image maps.

Image Map	Notation	Description
Reference Image	$I_{orig}$	photograph image to convert into bas-relief, given by smartphone camera
Original Depth Map	$D_{orig}$	depth map of $I_{orig}$ computed using multiview stereo reconstruction
Base Map	$D_{base}$	depth map representing overall shape of bas-relief, computed from $D_{orig}$
Detail Map	$D_{detail}$	depth map representing high-freq detail of bas-relief, computed from $I_{orig}$
DOF Depth Map	$D_{final}$	depth map computed by blending $D_{base}$ and $D_{detail}$ and DOF simulation

**Table 2:** Time for execution of each stage of bas-relief generation (unit: second).

Dataset	Size	Base Map Construction	Detail Map Construction	DOF Effects	Total
man1	384×512	2.67	5.26	2.51	10.44
man2		2.54	5.17	2.49	10.2
building	676×556	4.72	9.25	4.23	18.20
plamodel	384×512	2.32	4.91	2.31	9.54
